# Phantom-Based Evaluation of Scatter Radiation at Clinically Relevant Positions in Fluoroscopy-Guided Cardiac Procedures

**DOI:** 10.1016/j.jscai.2025.103936

**Published:** 2025-09-30

**Authors:** Robert F. Wilson, Paul Steege, Ashley Tao, Daniel Gomez-Cardona, John P. Gainor, Robert F. Riley, Jacob Kamen

**Affiliations:** a APIC Laboratory, University of Minnesota, Minneapolis, Minnesota; b Egg Medical, Inc., Arden Hills, Minnesota; c Diagnostic Physics and Radiation Safety Office, Gundersen Health System, La Crosse, Wisconsin; d Department of Cardiology, Overlake Medical Center, Bellevue, Washington; e Radiation Safety Office, Mount Sinai Health System, New York, New York

**Keywords:** fluoroscopy, scatter radiation, x-ray, x-ray guidance, x-ray phantom

## Abstract

**Background:**

Scatter radiation (SR) exposure to personnel performing fluoroscopy-guided procedures is significant and complex. With the development of next-generation shielding systems, it is essential to establish a standardized method of measuring SR for clinically relevant scenarios.

**Methods:**

A fixed C-arm x-ray unit was used to image the chest of an anthropomorphic phantom. SR was assessed using solid-state survey meters positioned at 6 positions where personnel typically stand during procedures. SR was measured from 20 to 200 cm from the floor at each position, in 5 commonly used radiographic projections. The radiation dose from the primary beam was measured below the table and at the image detector.

**Results:**

Of the primary x-ray beam, 96% was absorbed or scattered by the phantom. SR dose rates were markedly higher in positions around the head of the x-ray table (average x-ray dose rate at the 3 positions near the head 4.1 ± 0.6 fold the 3 positions away from the head, *P* < .01). X-ray angulation markedly increased SR dose compared to the anterior-posterior projection. The right anterior oblique and caudal views generated more SR than the left anterior oblique and cranial angulations (*P* < .01). SR intensity to the head, body, and leg regions varied significantly between positions around the table.

**Conclusions:**

Scatter radiation distribution around the x-ray table is highly dependent on the radiographic projection, with disproportionately high radiation dose levels around the head of the x-ray table and below it. This standardized method for measuring SR systematically may facilitate the assessment of enhanced radiation protection systems.

## Introduction

The use of fluoroscopically guided medical procedures has increased significantly with the development of minimally invasive techniques. These procedures are performed by hospital staff who are present in the room during the procedure and are exposed to scatter radiation (SR) from the patient and the x-ray unit. Although standard radiation shielding (eg, wearable shielding aprons and spot shields) is typically used by hospital personnel during these procedures, it offers limited protection, resulting in chronic, daily SR exposure, and has been associated with significant health risks.^1^, ^2^, ^3^, ^4^

More recently, advanced SR shielding systems have been developed. These systems have shown the potential to reduce or even nearly eliminate radiation exposure for hospital staff performing fluoroscopically guided medical procedures.^5^, ^6^, ^7^ However, an objective, systematic, and reproducible method to properly assess the effectiveness of these new shielding systems is important. A standard method for assessment of shielding efficacy in an experimental setting would allow comparisons of the relative effectiveness of systems designed to reduce SR.

Development of a standard method would need to mimic the clinical environment with an appropriate phantom and an x-ray system similar to that used clinically (both from the standpoint of configuration and with respect to the image acquisition settings). To effectively assess the performance of a shielding system, several factors need to be considered. First, SR is nonuniformly emitted from the patient and is therefore unequally distributed around the procedure room.^8^ Second, most fluoroscopically guided procedures involve imaging at different radiographic projections. The distribution of SR is known to change with angulation, and thus, measurements of scatter in these angulations must be included to be relevant.^9^ Third, personnel stand in different positions around the x-ray table depending on their role. Shielding effectiveness for one position might not be the same for another position, depending on the shielding system being tested. Therefore, measurements of SR at clinically relevant positions are essential to capture shielding effectiveness for all staff. Fourth, the phantom being imaged must reasonably replicate the SR distribution and energy spectrum present in a clinical environment. Finally, the protocol must include enough permutations of the factors previously mentioned to be comprehensive of the different scenarios; however, it must also be practical regarding the time and total number of measurements required.

In this study, we present a standardized method to measure SR from fluoroscopically guided procedures under clinically relevant conditions using an anthropomorphic phantom.

## Materials and methods

### Radiation measurement devices

Scatter radiation dose levels were measured using 6 solid-state survey meters (X2 Survey Sensor, Raysafe) that were all calibrated by the manufacturer within the prior 6 months. Our laboratory previously showed that the 95% confidence intervals of SR measurements using this meter are ±3.3%.^10^ Therefore, a single measurement per experimental condition was considered sufficient. SR dose levels were recorded as a dose rate (μSv/h).

Each survey meter was affixed to a holder mounted on a track in a calibrated pole, 2 m in height and oriented to the left chest, where the survey meter could be raised on the track with stops at every 20 cm (20-200 cm). This setup ensured that the angle and vertical height of the survey meter were easily reproducible between measurement conditions.

In order to measure the incident radiation dose from the x-ray primary beam for each radiographic projection, a solid-state x-ray dosimeter (X2 R/F Sensor, RaySafe) was used. The meter was placed right below the x-ray table, projected in the center of the imaging field, at the 4 corners of the field of view, and facing the x-ray tube. Incident radiation dose levels were recorded as a dose rate (μGy/s).

### X-ray imaging system

A fixed C-arm x-ray unit (2013 Toshiba Infinix, Toshiba America) equipped with a 12-inch flat panel detector was used. The unit was operated in fluoroscopic mode at 15 frames/s using the parameters shown in Table 1. X-ray parameters were controlled by an automatic brightness control (ABC) system that varied x-ray tube current (mA) and pulse width to achieve a prescribed level of image brightness. Tube voltage (kVp) remained unchanged at 70 kVp during ABC adjustments. The x-ray table height was set at 100 cm with an SID of 105 cm.

**Table 1:** X-ray unit parameters for different radiographic projections and incident radiation dose rates.

Angle	kV	mA	PW, ms	Air kerma rate, μGy/s	DRAP at table, mGy-cm^2^/s	DRAP at detector, mGy-cm^2^/s	DRAP detector/table, %	Scatter energy, keV
PA	70	65	6.6	192	21.5	0.916	4.3	40
RAO 30/cranial 20	70	85	7.6	275	23.2	0.744	3.2	42
LAO 30/cranial 30	70	58	6.3	157	15.0	0.831	5.5	39
RAO 25/caudal 20	70	138	9.9	617	65.4	0.184	2.8	43
LAO 30/caudal 20	70	66	6.7	197	33.4	0.302	0.9	42
Mean ± SD	70 ± 0	82 ± 29	7.4 ± 1.3	287 ± 169	31.7 ± 17.9	0.931 ± 0.51	3.3 ± 1.5	42 ± 2

DRAP, dose rate–area product; LAO, left anterior oblique; PA, anterior-posterior; PW, pulse width; RAO, right anterior oblique.

### X-ray phantom

We used a whole-body anthropomorphic phantom obtained from the U.S. Department of Energy Phantom Library (Model RESL 201).^11^ The anthropomorphic phantom, which was 178 cm in height, 25 cm chest thickness (anterior-posterior [PA]), and weighed 79 kg, generated SR that approximated a human.

### Experimental protocol

**Figure 1:**
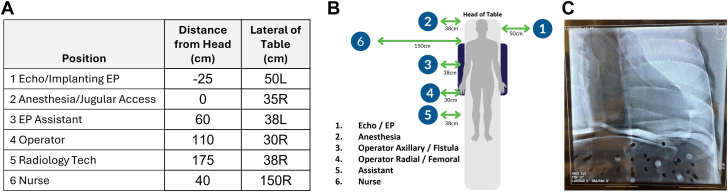
**Experimental setup with 6 measuring positions arrayed around the U.S. Department of Energy anthropomorphic phantom on a standard** x**-ray table.** (**A**) The measurement positions with respect to the x-ray table (L and R indicate left and right of the table edge, ± from the head indicates the distance from the top of the table). (**B**) A diagram of the measurement positions. (**C**) A representative x-ray image of the phantom.

The x-ray table with the anthropomorphic phantom was positioned such that the heart and the upper edge of the diaphragm were within a collimated 12-inch imaging field (Figure 1C). At each x-ray tube angulation, we recorded the radiation dose rate from the primary x-ray beam (air kerma from the x-ray unit, dose below the table, and dose at the image detector). This was to enable a comparison between the total measured SR with the actual output from the x-ray unit across different radiographic projections.

Scatter radiation measurements were taken simultaneously at 6 positions around the x-ray table, all corresponding to the positions typically used by the physician or staff during a procedure (Figure 1A, B). Position 1 was where an implanting electrophysiology (EP) physician or echocardiographer (for TEE imaging during structural procedures) stands. Position 2 was where an anesthesiologist might stand or where an operator might stand during jugular vein access procedures. Position 3 was where an assistant to the implanting EP physician would stand. Position 4 was where an operator would stand for radial or femoral access. Position 5 was where the assistant to the operator (the technician) would stand. Position 6 estimates where a nurse would typically stand for a procedure.

The survey meters were angulated such that the surface of the survey meter was aligned to the isocenter of the x-ray imaging system. In each condition, we obtained readings of SR at 20 cm intervals from the floor, starting at 20 cm and ending at 200 cm.

#### Experimental measurement conditions

We measured SR during imaging of the anthropomorphic phantom in 5 radiographic projections: (1) PA, (2) right anterior oblique (RAO) 30/cranial 20, (3) left anterior oblique (LAO) 30/cranial 30, (4) RAO 25/caudal 20, and (5) LAO 30/caudal 20.

### Data analysis

#### Dose from incident x-ray beam vs dose deposited on the image detector

To determine the fraction of the primary x-ray beam that reached the image detector, and therefore the fraction of the beam that was absorbed or scattered by the patient into the room, we measured the x-ray dose rate–area product (mGy⋅cm^2^/s) immediately under the table and at the detector. Because the dose on the detector may be inhomogeneous, we measured the dose rate at each of the 4 corners and at the middle of the detector, and then averaged all of the dose rates. The dose rate–area product at the detector was divided by the dose rate–area product under the table to determine the fraction of the x-ray beam that formed the image (ie, was not absorbed or scattered).

#### Two-dimensional distribution of SR in the horizontal plane

First, the average SR level for a given position and angulation was calculated by averaging all of the corresponding vertical radiation measurements. The average SR level for each position was also calculated by averaging all of the radiation vertical measurements across all 5 angulations. Finally, the average SR level for each angulation was obtained by averaging all of the radiation vertical measurements across all 6 positions.

#### Vertical distribution of SR around the x-ray table

To understand the distribution of SR below and above the x-ray table, the percentage of SR below the x-ray table was computed. This was achieved by grouping vertical measurements below the x-ray table and phantom (20-100 cm) and comparing them with those obtained above the x-ray table and phantom (120-200 cm).

The radiation exposure to the neck and head, body and thighs, and lower legs and feet (below a shielding apron) was computed by adding the vertical SR measurements corresponding to each area of the body (160-180 cm above the floor for the neck and head, 60-140 cm for the body and thighs, and 20-40 cm for the lower legs and feet).

Furthermore, an index of average room SR with each angulation was calculated by averaging all 10 vertical radiation measurements at all 6 positions for a given angulation.

Group values are presented as mean ± SD. Differences in SR dose levels between positions and angulations were tested using a paired *t* test, with a Bonferroni correction for repeated analyses. A *P* value < .05 was considered significant.

## Results

### X-ray parameters, incident radiation dose, and dose to the detector

X-ray system parameters and dose rates are shown in Table 1. The average radiation dose rate–area product at the imaging detector was 3.3% ± 1.5% of under the table dose rate–area product. Hence, an average of more than 96% of the primary beam was either absorbed by the phantom or scattered into the procedure room.

### SR using anthropomorphic phantom

#### Circumferential distribution of SR around the x-ray table

**Figure 2:**
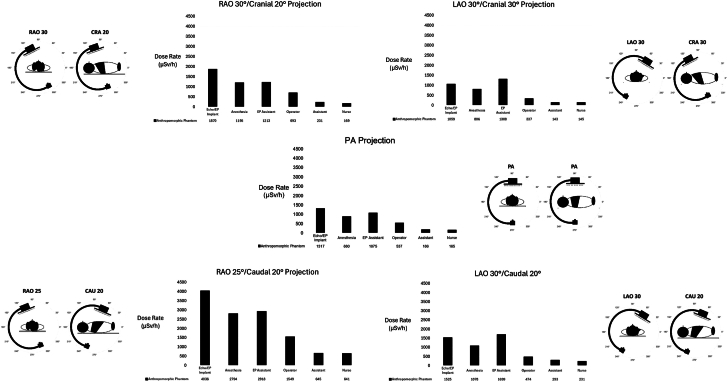
**Average scatter radiation dose levels (across all corresponding vertical measurements) for each position and x-ray tube angulation.** Right anterior oblique (RAO) and caudal (CAU) angulation resulted in the greatest scatter radiation dose levels across all positions. Positions near the head of the table consistently had higher radiation dose levels. CRA, cranial; LAO, left anterior oblique; PA, anterior-posterior.

In the PA projection, SR was highest in the positions near the head of the x-ray table and under the table (Figure 2, Tables 2 and 3). The ratio of radiation dose level of all heights and x-ray angulations in positions 1 to 3 (near the head of the x-ray table) was 4.1 ± 0.6 fold the average dose rates for positions 4 to 6.

**Table 2:** Average scatter radiation dose rate by position and x-ray tube angulation.

X-ray tube angle	Radiation dose by position, μSv/h						Average
	1. Echo/implanting EP	2. Anesthesia/jugular access	3. EP Assistant	4. Operator	5. Assistant	6. Nurse	
PA	1317	880	1075	537	186	165	693 ± 434
RAO 30/cranial 20	1870	1195	1213	693	231	169	895 ± 599
LAO 30/cranial 30	1059	806	1308	337	143	145	633 ± 453
RAO 25/caudal 20	4036	2794	2918	1549	645	641	2097 ± 1255^c^
LAO 30/caudal 20	1525	1078	1699	474	293	231	883 ± 586
Average	1961 ± 1061^a^	1351 ± 435^a^	1643 ± 571^a^	718 ± 431^b^	299 ± 180	270 ± 188	1040 ± 652

LAO, left anterior oblique; PA, anterior-posterior; RAO, right anterior oblique.

a *P* < .01 vs positions 4, 5, and 6.

b *P* < .01 vs positions 5 and 6.

c *P* < .01 vs all other angles.

**Table 3:** Percentage of scatter radiation dose measured below the x-ray table.

X-ray tube angle	Scatter radiation dose by position						Average
	1. Echo/EP implant	2. Anesthesia/jugular access	3. EP assistant	4. Operator	5. Assistant	6. Nurse	
PA	60%	74%	73%	70%	60%	56%	67% ± 7%
RAO 30/cranial 20	54%	82%	83%	68%	59%	66%	68% ± 11%
LAO 30/cranial 30	73%	67%	70%	48%	52%	67%	66% ± 13%
RAO 25/caudal 20	49%	76%	85%	82%	66%	62%	68% ± 12%
LAO 30/caudal 20	72%	37%	73%	68%	62%	60%	67% ± 6%
Average	61% ± 10%	67% ± 16%	77% ± 6%	67% ± 11%	60% ± 4%	62% ± 4%	67% ± 1%

LAO, left anterior oblique; PA, anterior-posterior; RAO, right anterior oblique.

**Figure 3:**
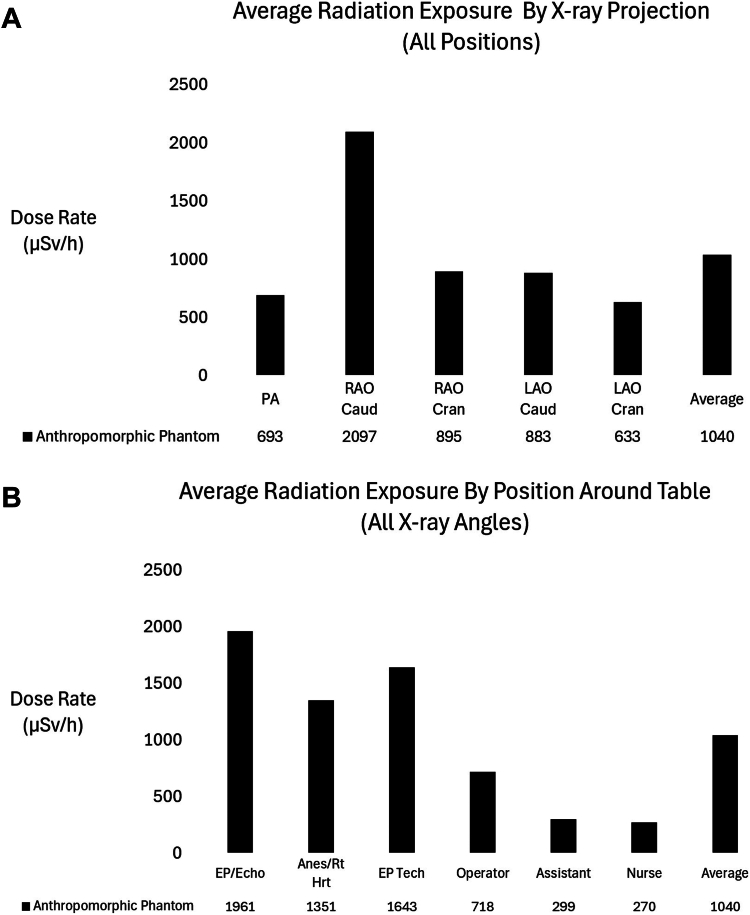
**Average room scatter radiation dose levels for each angulation across all personnel positions** (**A**). Average room scatter radiation dose levels for each position across all x-ray tube angulations (**B**). Right anterior oblique (RAO)/caudal (Caud) angulation had the highest average scatter radiation compared to all other angulations. The positions near the head of the table (positions 1-3) had the highest scatter radiation. Cran, cranial; LAO, left anterior oblique; PA, anterior-posterior.

**Figure 4:**
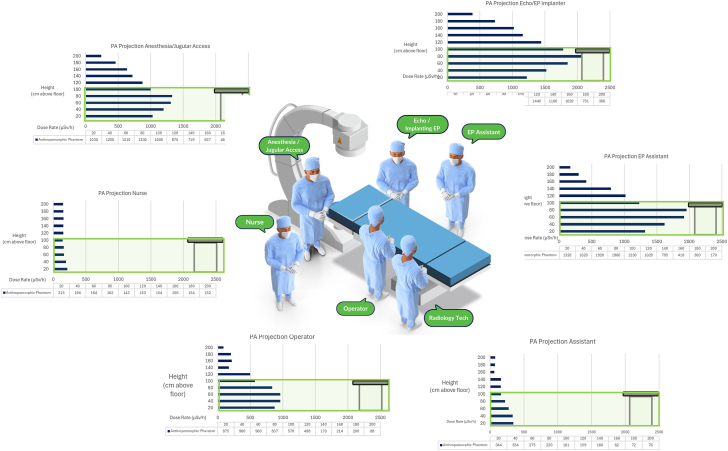
**Distribution of scatter radiation for the anterior-posterior (PA) radiographic projection and each of the 6 clinically relevant positions.** The x-ray table and shaded area are drawn for reference.

With x-ray angulation, the intensity of SR increased significantly (except in the LAO cranial angle), but the positions near the head of the table still exhibited the highest SR intensity (Figures 3 and 4). Positions 1 to 3 around the head had an average SR intensity in the angulated views of 1651 μSv/h compared to 429 μSv/h for positions 4 to 6 (*P* < .01).

**Figure 5:**
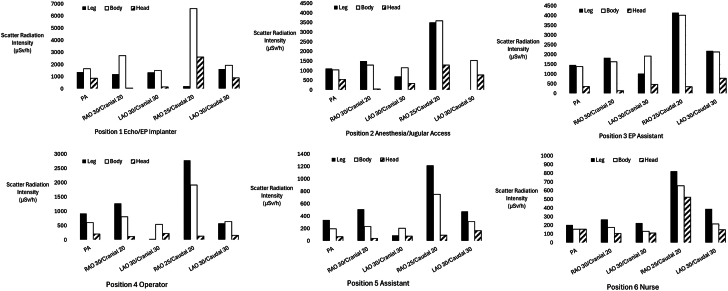
**Scatter radiation dose levels for the head, body, and legs, plotted by the position around the table for each x-ray projection (note the scale differences between graphs for clarity).** LAO, left anterior oblique; PA, anterior-posterior; RAO, right anterior oblique.

In all positions around the table, the RAO Caudal angulation produced the most intense SR dose (average SR 2097 ± 1255 μSv/h compared to 693 ± 434 μSv/h in the PA projection, *P* < .01). Complete details of the SR intensity with x-ray beam angulation can be seen in Table 2 and Figures 3 to 5. Generally, RAO angulation produced more SR around the table than LAO projections. Caudal angulations produced more SR than cranial angulations.

#### Vertical distribution of SR around the x-ray table

Scatter radiation intensity was markedly higher below the x-ray table, regardless of angulation. In the PA projection, 67% ± 7% of the SR was directed below the table and 33% ± 8% was directed above the table (Figure 2 and Table 3).

Angulation significantly increased SR above and below the table, but the relative increase with each angle was dependent on the position. Figure 5 shows the radiation dose levels that a 180 cm tall person would receive to the head and neck, body and thighs, and lower legs and feet for each position around the x-ray table for different radiographic projections. The Central Illustration expands these results specifically for the PA projection. We divided exposures into these groups because the standard use of shielding aprons would limit body irradiation, but exposure to the head, arms, and legs would persist.

**Central Illustration:**
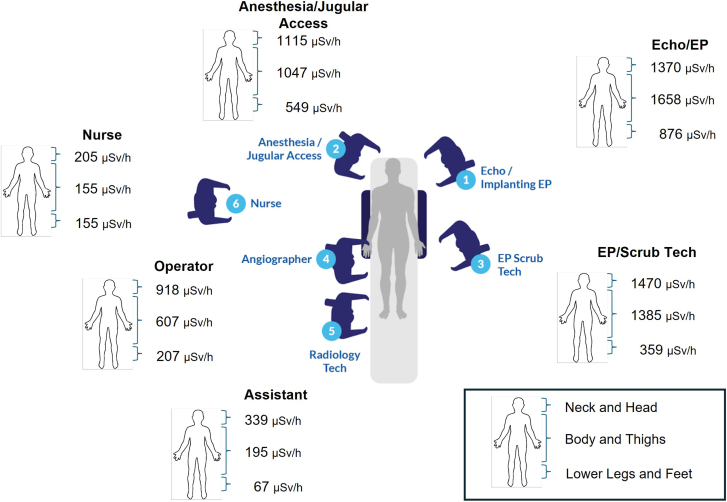
**Scatter radiation dose levels averaged by body part and displayed for all positions around the table for theanterior-posteriorprojection.** EP, electrophysiology.

With x-ray tube angulation, the SR dose levels to most but not all body areas at all positions increased markedly. The distribution of radiation scatter for each body area depended significantly on the personnel’s position and radiographic projection. The SR dose levels for the leg were typically higher for all positions, regardless of the projection; only for position 1 (echocardiographer or EP implanter) was the dose to the body higher than that of the leg. In part, this may be due to the fact that the body dose was very high in positions 1 to 3 compared to positions 4 to 6 farther down the table. Of note, even in the PA projection, the head exposure in position 1 was 1370 μSv/h compared to 918 μSv/h for the operator in position 4.

## Discussion

These studies emphasize the nonuniform nature of human SR. Over two-thirds of SR was distributed below the table, and the area around the head had 4-fold the radiation intensity compared to the operator, assistant, and nurse positions. Angulation markedly increased SR. In general, SR was greatest in the RAO and caudal angulations.

Accurate spatial information about the SR cloud that floods the procedure room where fluoroscopically guided procedures are performed is important to: (1) improve the radiation protection of staff involved in these procedures, and (2) assess the effectiveness of novel shielding systems. Unfortunately, detailed measurements of SR during clinical procedures are difficult. Radiation exposure times are relatively short, and x-ray gantry angulation is frequent, making the collection of detailed spatial data nearly impossible. Therefore, there is a need for a standardized phantom-based protocol to assess the SR distribution in a clinically meaningful way.

There have been many studies that previously evaluated the extent and distribution of SR.^12^, ^13^, ^14^ Phantom-based studies have used a variety of sampling positions, often limited in number and without a great deal of spatial resolution. Spatial resolution is important because angulated views are integral to clinical practice; many studies report measurements with limited or no x-ray angulation. Clinical studies where cumulative case dosing is measured completely mimic the clinical environment, but the cumulative SR dose is dependent on the patient dose–area product and the specific angulations of each case, making comparisons between shielding systems difficult.

In collecting data using this method, we attempted to provide clinically relevant measurements by measuring SR at 6 points around the x-ray table where personnel typically stand. We showed through the vertical measurements of radiation doses at these positions that body areas not typically covered by a standard shielding apron (head, lower legs, and arms) have substantial radiation exposure. Although additional shielding, such as table shielding and hanging shields, may attenuate the radiation doses to these areas for the operator, other personnel in the room have minimal ancillary shielding. This underscores the importance of adequate shielding to protect highly irradiated areas of the body not covered by a shielding apron, particularly for positions in the room not covered by traditional shielding.

Finally, we measured the SR pattern in 5 x-ray angulations that are typical of most clinical procedures. As these data show, x-ray angulation not only generally increases the intensity of SR but it also changes the circumferential and vertical distribution.

Our findings are consistent with recent reports of excessive SR exposure for personnel near the head of the x-ray table.^15^^,^^16^ Transesophageal echocardiographic imaging during structural heart procedures is associated with a marked increase in personnel SR exposure, especially in RAO projections. Similarly, exposure during procedures using jugular vein access exposes the operator to excessive SR dose, similar to the operator dose during coronary angiography, despite the procedures being performed with minimal or no x-ray tube angulation and the relative brevity of the procedures.

Finally, we assessed the efficiency of fluoroscopic imaging—the fraction of the primary beam that contributes to forming the image compared to the fraction that is absorbed or scattered. Using the x-ray unit in this study, we found that far less than 4% of the primary x-ray beam was deposited on the image detector, meaning that well over 96% of the x-ray photons are absorbed by the patient or scattered in the room. It is possible that the efficiency of other x-ray units is better. In addition, the use of image processing software may allow further reductions in primary beam intensity.^17^ Despite these reductions in air kerma needed to obtain an acceptable image, the relative distribution of SR around the procedure room should be similar, just lower in intensity.^18^ Further study using additional x-ray units with advanced image processing will be helpful.

Management of the x-ray tube output through the ABS system varies among vendors, and the algorithm is often proprietary.^19^ The unit we used primarily increased the number of photons reaching the detector by increasing the tube current (mA) and pulse width; tube voltage was held at 70 kVp. It is possible that different algorithms or tube settings would generate differences in SR dose levels and perhaps even the spatial distribution of such scatter. Additional studies examining the effects of various ABC settings will help define the parameters that provide acceptable imaging with the minimum of SR generation.

Our study does have several limitations. We only studied chest imaging. Chest imaging typically produces less SR than abdominal imaging due to the overall lower x-ray attenuation provided by the composition of the chest. The chest, however, is less radiographically homogeneous than the abdomen. The radiodensity of the chest varies markedly with x-ray tube angulation, depending on the angle and the ratio of air-filled lung to soft tissue in the imaging field. In addition, we studied SR related to heart and chest imaging. The pattern and intensity of SR from imaging of different body areas will require further study.

We also studied only one anthropomorphic phantom size. Although this might be appropriate for a standard method to assess shielding systems, the absolute SR intensities vary with the size of the x-ray target. A prior study showed that the amount of SR developed during clinical studies varied significantly with patient body mass index.^20^ Other full-body phantoms might also be useful, provided they generate sufficient scatter comparable to a human and they provide a similar scatter pattern.

In conclusion, we present a standardized method for evaluating SR, which could be utilized to evaluate next-generation shielding systems that have recently been introduced into clinical practice. Development of standardized methods for assessing SR protection at clinically relevant personnel positions and using x-ray tube angulations that replicate the clinical procedure should facilitate the design and assessment of these new protection systems.
